# Supplementary material for: Enablers and barriers to community pharmacists’ readiness to implement deprescribing of inappropriate medications for older adults in Qatar
Source: PLoS One. 2025 Jan 30;20(1):e0316363. doi: 10.1371/journal.pone.0316363 (PMC11781633; doi:10.1371/journal.pone.0316363)
Supplement: Attachment — Submitted filename: PLOS ONE ReviewFINAL Author Editor.pdf [file pone.0316363.s001.pdf]

**PLOS ONE Review**

**TITLE: Enablers and barriers to community pharmacists' readiness to implement deprescribing of inappropriate medications for older adults in a developing setting**

For greater clarity, please amend "… in a developing setting" to in Qatar.

The use of some terms such as developed and developing can be fraught since it can be challenging and even problematic to try to divide all the world's nations into one of two categories. The different status is stated to represent nations that may be agrarian, resource-poor, low income/low living standard as developing compared to nations that are industrialised, wealthy and/or resource rich, with developing nations able to access development aid. In December 2022, 80 nations globally were considered to be high income nations whilst 137 were considered to be developing economies (https://www.developmentaid.org/news-stream/post/154288/how-do-developed-countries-differ-from-the-developing-ones). The terms can be fraught also as while the United Nations (UN) uses the developed/developing delineation, the World Bank uses different categories, these being upper-middle; lower-middle and low-income countries. The challenge with a nation such as Qatar is that GDP per capita in 2024 is calculated as $81,400 by the IMF expressed in US dollars (https://www.imf.org/external/datamapper/profile/QAT). When comparing with other nations, Qatar is identified as fifth richest nation in the world in 2024 (https://gfmag.com/data/richest-countries-in-the-world/). Further, the poverty rate for Qatar is stated to be across 2019-2024 at 0.0% (https://www.macrotrends.net/global-metrics/countries/QAT/qatar/poverty-rate). So, rather than categories, identifying the name of the country and location can be more helpful. As an example, if a researcher wanted to know whether the colour of a road might have an impact on global warming, then being able to access data from Qatar would be really important as the blue roads are proposed to decrease temperatures the asphalt by 15 - 20 degrees Celsius (https://www.roadmarkingqatar.com/post/why-blue-colour-roads-in-qatar).

**RELEVANCE OR SIGNIFICANCE:** Currently those in Qatar older than sixty years of age constitute 3.6% of the population though that is predicted to increase to approximately 20.3% by 2050, which is in 26 year's time, so lacking apparent immediate urgency. So, seek to identify the significance or implications of undertaking this study now in 2024. There is time available to review and amend pre-registration qualifications offered in Qatar by adding or increasing medicines simplification, quality use of medicines and de-prescribing content. In addition, there is time to develop continuing professional development materials, and propose credentialling or recognised qualifications to registering authorities.

**KEY WORDS: Currently-** Community pharmacists, deprescribing, older adults, enablers, barriers. There is mention of training needs, education enhancement in the

manuscript so perhaps adding : curriculum review, continuing professional development

**ETHICS:** Ethics approval was obtained from QUIRB (Qatar University Institutional Review Board) (QU-IRB 1906-E/23, August 30, 2023).

**ABSTRACT:** (268-word count) PLOS ONE requires an abstract to: describe the main objective(s) of the study, explain the study (what was done); summarize the results and their significance; not exceed 300 words.

The abstract identified the aim of the study, briefly characterised the study respondents, perceptions of barriers and enablers as elicited and implications of the results for the future.

***Some enhancements:***

Line 31 The current study *assessed determined*... It is possible to use one or other word

Lines 35-*36 "Enablers and barriers were assessed with a 5-point Likert-type scale"*. Something such as - Information about perceptions of enablers and barriers were elicited using a 5-point Likert scale - This provides additional clarity as to the purpose and what was disclosed by respondents.

**INTRODUCTION:** PLOS ONE expectations for introductory material is for: provision of sufficient background and context of the manuscript such that readers are able to understand the purpose and significance of the study; clarity in identifying the issue addressed and why it is problematic or necessary; concluding with a brief description of the overall aim and whether/how that was/was not achieved. Comments on this section of this manuscript follow.

Lines 57-59. Deprescribing is identified as "... an important clinical tool..." but for clarity, it would be helpful to offer a definition or detailed description of deprescribing as used in this study. Although deprescribing as a term was stated to be first used in the English language health literature in 2003 in an Australian hospital pharmacy journal, over subsequent years the term has been customised, for example in patients taking cardiovascular medicines only, and the process to some extent modified(Reeve, E., Gnjidic, D., Long, J., & Hilmer, S. (2015). A systematic review of the emerging definition of 'deprescribing' with network analysis: implications for future research and clinical practice. British journal of clinical pharmacology, 80(6), 1254–1268. https://doi.org/10.1111/bcp.12732) , so clarity in this study's use is beneficial.

Lines 63-65 assert that the greatest risk to the elderly from medication lies in the number of medicines, and certainly, the more medicines taken, the more there can be a risk of drug-drug, drug-herb, drug-disease state interactions, but there are also some medicines that have an inherent risk of adverse effects in the elderly, even with a lesser number of medications taken. These include NSAIDS, medicines with an anticholinergic

effect, some diuretics, antihypertensives and others (https://www.msdmanuals.com/en-au/professional/geriatrics/drug-therapy-in-older-adults/drug-categories-of-concern-in-older-adults).

Lines 76-78- Currently, “Furthermore, Martin et al, in a cluster randomized controlled trial among older adults in Canada reported that community pharmacists-led deprescribing resulted in *greater discontinuation* of inappropriate medications.” So, what is the comparator- Greater than prescribers? Greater than the patient’s requests? This benefits from additional clarity as to the insights relevant to or which informed this study.

Lines 85-88. – Currently, “The enabling factors include the use of tailored deprescribing guideline within a structured multidisciplinary framework, use of a multidisciplinary deprescribing model involving physicians, pharmacists and nurses, effective interprofessional collaboration…”. These statements are referenced in the paragraph with references 14-16. Readers seeking more information perhaps of an unfamiliar term such as “a structured multidisciplinary framework” or an unfamiliar procedure such as “… use of a multidisciplinary deprescribing model…” would benefit from each key assertion being individually referenced, especially as these are identified as enabling factors.

Lines 92-98. Likewise, the benefit of additional clarity for the barriers.

Lines 102-104. The manuscript states, “Indeed, only one such study focused on the assessment of community pharmacists’ knowledge of deprescribing and their self-perceived enablers and barriers to providing the service in the United Arab Emirates was identified.” It can be helpful to identify the search criteria utilised as a quick search identified four studies that may have been helpful, two studies in 2024 in Nigeria (exploring barriers and enablers), one in Malaysia in 2023(deprescribing) and also in 2023 the study cited in reference 20 (key to promoting deprescribing).

Lines 112-117. The manuscript states, “The current study was foregrounded by the Theoretical Domains Framework (TDF) V2 [21]. This is a theoretical framework that assesses the factors that may enable or militate against the successful implementation of an intervention focused on a specific desired practice change. The TDF consists of fourteen domains developed from 128 theoretical constructs that were synthesized from varieties of behavioural change theories associated with implementation science and practice change…” It could assist readers to identify additional information such as the fourteen domains and those especially relevant to this study, for example.

**METHODS:** The requested information in PLOS ONE is detailed and clear – the journal requires sufficient detail to allow suitably skilled researchers to fully replicate this study. When the methods are well established, authors may cite articles where those are described in detail, but even so this manuscript should include sufficient information to be understood independent of those references.

Lines 140-144. Please see comment above seeking the fourteen domains and those most relevant to this study. It is stated the fourteen domains were grouped into 5 categories, were these grouped by the research team or is this grouping inherent in the TDF? It goes on to say this grouping was influenced by an Irish study but would an Irish study and a Qatari study benefit from the same/similar categorisation? Please explain the research team's decision-making on this point. Please describe the fourteen domains and the theoretical background as it informs this study. In which geographical region was the framework developed? How does it apply to Qatar?

Lines 159-160. For clarity please, does this mean that the responses from the pre-test sample were not included in the dataset of the study for analysis? What happened to them?

Lines 170-173. The three research assistants, were these individuals, students, administrative support people or pharmacists? This section could be enhanced by some details about the research assistants since they seem to be the "face" of this study to pharmacist participants. This is a concern because response rates for participation and completion can vary with the characteristics of the person providing the questionnaire. Back in 2002, it was established that even the colour of the paper a printed questionnaire was printed on made a difference, Etter and colleagues, for example, established that pink paper increased response rates by 12%, so provision of detail is important.

Line 173. The detailed "information about the purpose and anticipated benefits of the study," how was the information provided? Verbally, written, link to study website? Additional information will assist readers as they consider their own studies.

Lines 176-177. Please identify by whom they were collected. Research assistants again? The same research assistants for each of the research participants?

**ANALYSIS:** The manuscript identifies that IBM SPSS (Statistics for Windows, Version 29.0. Armonk, NY: IBM Corp.) software was used for data analysis. Descriptive statistics such as median (IQR) was used for continuous data with non-normal distribution while frequency (%) was used for categorical data, with significance set at $p \le 0.05$.

In a manuscript, it is beneficial to identify those functions within SPSS that were used to analyse the data, similarly any recoding of values such as for negatively phrased items. So, was the descriptive function used? Was Factor analysis? If it was then the approach is crucial so PAF, PCA, exploratory? Perhaps Cross-Tabulations might have been used to explore a relationship between two or more variables? More detailed information is needed to understand the results obtained and the conclusions drawn. In Methods it was stated that, "...23-item questionnaire comprising three sections including A (demographics = 10 items), B (Enablers = 7 items), and C (Barriers = 6 items). ", so, an

expectation would be that how each section was analysed would be clearly identified. Further, study participants were asked to rank their responses to the items in Section B and C on a 5-point Likert-type scale (highest =5, high = 4, moderate = 3, low = 2, Lowest = 1). However, in Results section Table 2, the columns are labelled,

| Low<br><br>n (%) | Moderate<br><br>n (%) | High<br><br>n (%) | Median (IQR) |
|---|---|---|---|

If additional analyses or re-coding were undertaken to result in these three categories, that needs to be clearly detailed. Likert data may also be presented as histograms or bar charts where data can be visualised.

Lines 173- 176 state, "The respondents who agreed to participate in the study signed the written informed consent form before the start of data collection, and they were all informed of their right to decline participation at any point in the study." As does Lines 165-167, "The respondents who agreed to participate in the study signed the written informed consent form before the start of data collection, and they were all informed of their right to decline participation at any point in the study." Please delete one occurrence as these sentences are closely situated and one becomes redundant.

**RESULTS:** PLOS ONE accepts single categories across Results/Discussion/Conclusions or merged categories such as Results/Discussion or Discussion/ Conclusions. This manuscript presents all three sections separately. Only Results will be addressed for review in this section.

The Results section starts with a presentation of the characteristics of the respondents noting for example, that "… [they] consisted mainly of 5 nationalities (91.0%; 182/200) including Indian (40.5%), Egyptian (22.5%), Jordanian (22%) and Sudanese (6%). Since only 182/200 may have responded to this item the absence of Qatari respondents was of interest as in 2024 it might be expected that there could be perhaps some 12 cohorts of graduating students from Qatar University since the course is listed as commencing in 2007 with full accreditation in 2011 (https://www.qu.edu.qa/static_file/qu/colleges/pharmacy/documents/Pharmacy%20A4%20Magazine-compressed%2019AY.pdf).

Additional detail would be helpful to understand the results of the study and its outcomes.

The sociodemographic profile of the respondents was featured also in Table 1 which established that data from ten nationalities in total was included. With all nationalities included, the respondent total is 200, the total number of listed respondents.

The next results reported are the results for the seven enabling factors and separately the seven factors that act as barriers to community pharmacists' readiness to implement deprescribing for older adults in Qatar. The items were presented individually, with responses rated as low, medium or high by number of respondents and the per centage that represents. It would be helpful to know what numerical values were represented by the categories, low, medium and high so please provide these. It was helpful to see the item wording and would also have been beneficial in the methodology section because what an item seeks to elicit varies by the wording used. Indeed, the exact wording of all items actually presented to respondents benefits by being available in Methods. Additional guidance is available in this article, "A Consensus-Based Checklist for Reporting of Survey Studies (CROSS)" in which the reporting guidelines are found in the supplementary material – supplement 3

(Sharma, A., Minh Duc, N. T., Luu Lam Thang, T., Nam, N. H., Ng, S. J., Abbas, K. S., Huy, N. T., Marušić, A., Paul, C. L., Kwok, J., Karbwang, J., de Waure, C., Drummond, F. J., Kizawa, Y., Taal, E., Vermeulen, J., Lee, G. H. M., Gyedu, A., To, K. G., Verra, M. L., ... Karamouzian, M. (2021). A Consensus-Based Checklist for Reporting of Survey Studies (CROSS). Journal of general internal medicine, 36(10), 3179–3187. https://doi.org/10.1007/s11606-021-06737-1).

**DISCUSSION:** The discussion identified that the community pharmacist respondents would find continuing professional development, the availability of a shared electronic patient record and an improved remuneration package to be key enabling factors in deprescribing in older adults in Qatar who currently reported to be 3.6% of the population. However, there are already reports of an initiative in outpatient pharmacies at Rumailah Hospital, Qatar (Alyazeedi, A., Sherbash, M., Algendy, A. F., Stewart, C., Soiza, R. L., Alhail, M., Aldarwish, A., Stewart, D., Awaisu, A., Ryan, C., & Myint, P. K. (2024). Enhancing Medication Safety through Implementing the Qatar Tool for Reducing Inappropriate Medication (QTRIM) in Ambulatory Older Adults. Healthcare (Basel, Switzerland), 12(12), 1186. https://doi.org/10.3390/healthcare12121186). If hospital outpatient pharmacies in 2022-23 are using, and others perhaps intending to use Qatar Tool for Reducing Inappropriate Medication (QTRIM) in Ambulatory Older Adults i.e. community dwelling, what is the rationale for community pharmacists to also undertake deprescribing? This considers the significance of the outcomes of the manuscript study compared to an existing tool and implementation in outpatient pharmacies. Please discuss and contextualise also with this statement in the manuscript, "... Furthermore, the identification of the regulatory framework currently guiding community pharmacy practice in Qatar as a key barrier to the implementation of deprescribing in practice is being reported for the first time. This is because the range of clinically-oriented services that community pharmacists are legally permitted to provide are limited..."

It may also be helpful to consider for this manuscript, any potential influence of the discussion on inappropriate polypharmacy management as contrasted to deprescribing in a 2023 publication by Zaidan and Awaisu.

**CONCLUSIONS :** The conclusions section of the manuscript revisits the enablers and concludes that implementation of deprescribing may be feasible, noting however that the barriers likely will require significant educational, legislative, and practice changes.

For enhancement, in this section, CPD is desired as an enabler as roughly half the respondents did not learn deprescribing as students, who is then qualified to teach this? Might not another barrier be development of training for registered pharmacists and curricular inclusion for students? This may take significant time, consultation with key stakeholders, possibly even the formation of a collaborative pharmacist professional body to agree on those learning activities that are mandated and those that may be optional electives such as "specialist" areas diabetes, cardiovascular disease, for example. It also important to know which town or towns were sampled as different locations may hold different views or contrary views. It would also be beneficial to have estimates of desired achievements or deliverables, so for example, at year 1, year 3, year 5 and year 10.

**LIMITATIONS:** Limitations section identifies those characteristics of the study methodology or design that impact the research outcomes or interpretations. As such the limitations are addressed in the section from lines 294 to 301. The limitations identified were firstly, non-probability sampling as participants were drawn predominantly from chain or banner group pharmacies which represent 75% approximately of the community pharmacies in Qatar, however it was not evident in the manuscript which locations were sampling towns or regions as differences in perceptions, experiences and resources might be anticipated in various locations. So, this is a valid issue and benefits from discussion as to any ameliorating factors or processes that the research team may have applied. It was also not known how community pharmacies are owned as the owners may influence pharmacists' views.

The second limitation noted was that a quantitative survey design may have triggered social desirability bias or social acquiescence though the link between methodology (survey) and favourable responses is not clearly explained but benefits from being so.

**REFERENCES:** There are thirty-seven references cited in the manuscript of which six are aged (>10 years) with eleven aged within the last five years, for recency. Vancouver style is listed as the expectation of PLOS ONE.

This reference (number 36) appears to be inconsistent with Vancouver referencing:

Gerlach N, Michiels-Corsten M, Viniol A, et al. Professional roles of general practitioners, community pharmacists and specialist providers in collaborative

medication deprescribing—a qualitative study. BMC Fam Pract. 2020/09/06 ed. 2020 Sep 4; 21(1):183.

Please review the reference list for consistency with style.
