# Supplementary material for: Enablers and barriers to community pharmacists’ readiness to implement deprescribing of inappropriate medications for older adults in Qatar
Source: PLoS One. 2025 Jan 30;20(1):e0316363. doi: 10.1371/journal.pone.0316363 (PMC11781633; doi:10.1371/journal.pone.0316363)
Supplement: Attachment — Submitted filename: Response to Reviewers.docx [file pone.0316363.s002.docx]

29 October 2024

The Editor-In-Chief

PLOS ONE

Dear Sir,

Re: Manuscript #PONE-D-24-26224 – “Enablers and barriers to community pharmacists’ readiness to implement deprescribing of inappropriate medications for older adults in a developing setting”

Our sincere thanks for the opportunity to revise the manuscript #PONE-D-24-26224, titled “Enablers and barriers to community pharmacists’ readiness to implement deprescribing of inappropriate medications for older adults in a developing setting” which is under your consideration for publication in the PLOS ONE. We thank the reviewers for the insightful comments and useful suggestions and we have revised the manuscript accordingly. Please find stated below our point-by-point response to the reviewers’ comments. We have also revised the manuscript in accordance with the editor’s comments.

**EDITOR’S COMMENTS**

Journal Requirements:

1. When submitting your revision, we need you to address these additional requirements. Please ensure that your manuscript meets PLOS ONE's style requirements, including those for file naming. The PLOS ONE style templates can be found at https://journals.plos.org/plosone/s/file?id=wjVg/PLOSOne_formatting_sample_main_body.pdf and

https://journals.plos.org/plosone/s/file?id=ba62/PLOSOne_formatting_sample_title_authors_affiliations.pdf

**Response:** The corrections have been done in accordance with specifications stated in the PLOS ONE style template

2. Thank you for stating the following financial disclosure:

KBY

Undergraduate Research Experience Program (UREP) award [UREP29-092-3-029] from the Qatar National Research Fund (a member of The Qatar Foundation). The contents are solely the responsibility of the authors. Please state what role the funders took in the study. If the funders had no role, please state: "The funders had no role in study design, data collection and analysis, decision to publish, or preparation of the manuscript." If this statement is not correct you must amend it as needed. Please include this amended Role of Funder statement in your cover letter; we will change the online submission form on your behalf.

**Response:** The financial disclosure statement has been amended as recommended, and also included in the revised cover letter.

3. Please amend either the abstract on the online submission form (via Edit Submission) or the abstract in the manuscript so that they are identical.

**Response:** The abstract has been amended as recommended.

**REVIEWER’S COMMENTS**

**Reviewer #1:** The manuscript as presented could benefit from additional information, rationale to support decision-making and clarity. These follow in summary and more detail in peer review report.

Response: We are truly grateful for the valuable comments offered by the reviewer and the time spent to provide these useful feedback. We value the suggested corrections proposed by the reviewer and we are convinced it can only improve the scholarly value of the manuscript.

- **Comment-1**: Amend title to include Qatar.

**Response-1:** We thank the reviewer for this suggestion and we totally concur with the premise of the suggested correction. The title has been revised to include “Qatar” [Pg 1, line 2].

- **Comment-2:** Currently those in Qatar older than sixty years of age constitute 3.6% of the population though that is predicted to increase to approximately 20.3% by 2050, which is in 26 years’ time, so lacking apparent immediate urgency. So, seek to identify the significance or implications of undertaking this study now in 2024. There is time available to review and amend pre-registration qualifications offered in Qatar by adding or increasing medicines simplification, quality use of medicines and de-prescribing content. In addition, there is time to develop continuing professional development materials, and propose credentialing or recognized qualifications to registering authorities.
- **Response-2:** We thank the reviewer for the important observation and we see the points raised. However, we are of the opinion that the potential clinical and financial burden associated with the harms inherent in the use of inappropriate medications in elderly population is better avoided by provision of a clinical service such as deprescribing especially at the primary care level where community pharmacists are easily accessible. In addition, 3.6% of the current population translates to about 120,000 elderly patients in Qatar (Estimated pop is about 3million). Hence, there is a lot of potential inherent benefits associated with the deployment of a care-enhancing clinical service such as deprescribing of inappropriate medications in one of the most vulnerable patient group such as the elderly. In addition, it is generally well established that early deployment of interventions focused improving patient outcomes and strengthening the health system often result lasting positive impact on public health.
- **Comment-3:** KEY WORDS: Currently- Community pharmacists, deprescribing, older adults, enablers, barriers. There is mention of training needs, education enhancement in the manuscript so perhaps adding : curriculum review, continuing professional development.

**Response-3**: This is an excellent suggestion and we sincerely thank the reviewer. The revision has been done as recommended [Pg 3, line 47-48].

- **Comment-4:** Line 31 The current study assessed determined… It is possible to use one or other word.
- **Response-4:** Heartfelt thanks to the reviewer. This was an unintended error and it has been corrected [Pg 2, line 24].
- **Comment-5:** Lines 35-36 “Enablers and barriers were assessed with a 5-point Likert-type scale” . Something such as - Information about perceptions of enablers and barriers were elicited using a 5-point Likert scale - This provides additional clarity as to the purpose and what was disclosed by respondents.
- **Response-5:** Many thanks to the reviewer for the suggestion. The corrections have been done [Pg 2, line 28].
- **Comment-6:** Lines 57-59. Deprescribing is identified as “… an important clinical tool…” but for clarity, it would be helpful to offer a definition or detailed description of deprescribing as used in this study. Although deprescribing as a term was stated to be first used in the English language health literature in 2003 in an Australian hospital pharmacy journal, over subsequent years the term has been customised, for example in patients taking cardiovascular medicines only, and the process to some extent modified(Reeve, E., Gnjidic, D., Long, J., & Hilmer, S. (2015). A systematic review of the emerging definition of 'deprescribing' with network analysis: implications for future research and clinical practice. British journal of clinical pharmacology, 80(6), 1254–1268. https://doi.org/10.1111/bcp.12732) , so clarity in this study’s use is beneficial
- **Response-6:** Heartfelt thanks to the reviewer for this valuable comment. We agree that a definition of deprescribing will further enhance clarity. In fact, we respectfully submit that we have already done this in the last sentence of the first paragraph of the Introduction section. However, this sentence has been revised to enhance its clarity as recommended by the reviewer. In addition, we have also added one of the references kindly suggested by the reviewer because we think its definition of deprescribing seems to have been captured in line 61-65 [Pg 3-4]
- **Comment-7:** Lines 63-65 assert that the greatest risk to the elderly from medication lies in the number of medicines, and certainly, the more medicines taken, the more there can be a risk of drug-drug, drug-herb, drug-disease state interactions, but there are also some medicines that have an inherent risk of adverse effects in the elderly, even with a lesser number of medications taken. These include NSAIDS, medicines with an anticholinergic effect, some diuretics, antihypertensives and others (https://www.msdmanuals.com/enau/professional/geriatrics/drug-therapy-in-older-adults/drug-categories-of-concern-in-older-adults).
- **Response-7:** Sincere thanks to the reviewer for this excellent comment. We concur with the reviewer point and we think this assertion has already been captured in the manuscript [Line 61-65]. We were conscious of balancing comprehensiveness with conciseness to minimize redundancies.
- **Comment-8** Lines 76-78- Currently, “Furthermore, Martin et al, in a cluster randomized controlled trial among older adults in Canada reported that community pharmacists-led deprescribing resulted in greater discontinuation of inappropriate medications.” So, what is the comparator- Greater than prescribers? Greater than the patient’s requests? This benefits from additional clarity as to the insights relevant to or which informed this study.

**Response-8:** Sincere thanks for this important comment. The correction has been done to enhance clarity [Line 73-74].

- **Comment-9:** Lines 85-88. – Currently, “The enabling factors include the use of tailored deprescribing guideline within a structured multidisciplinary framework, use of a multidisciplinary deprescribing model involving physicians, pharmacists and nurses, effective interprofessional collaboration…”. These statements are referenced in the paragraph with references 14-16. Readers seeking more information perhaps of an unfamiliar term such as “a structured multidisciplinary framework” or an unfamiliar procedure such as “… use of a multidisciplinary deprescribing model…” would benefit from each key assertion being individually referenced, especially as these are identified as enabling factors.

**Response-9:** Many thanks to the reviewer and we see the point. However, for the sake of brevity, we cited the references at the end of the sentence. We think this is appropriate and consistent with the established norm in the scholarly world.

**Comment-10** - Lines 92-98. Likewise, the benefit of additional clarity for the barriers.

**Response-10** - Many thanks to the reviewer and we see the point. However, for the sake of brevity, we cited the references at the end of the sentence. We think this is appropriate and consistent with the established norm in the scholarly world.

- **Comment-11:** Lines 102-104. The manuscript states, “Indeed, only one such study focused on the assessment of community pharmacists’ knowledge of deprescribing and their selfperceived enablers and barriers to providing the service in the United Arab Emirates was identified.” It can be helpful to identify the search criteria utilised as a quick search identified four studies that may have been helpful, two studies in 2024 in Nigeria (exploring barriers and enablers), one in Malaysia in 2023(deprescribing) and also in 2023 the study cited in reference 20 (key to promoting deprescribing).
- **Response-11:** Many thanks for the interesting suggestion. The studies alluded to in the review comments that were conducted in Nigeria and Malaysia were done among HCPs including physicians, hospital pharmacists, nurses practicing in secondary and tertiary care settings. Our study was focused mainly on community pharmacists.
- **Comment-12:** Lines 112-117. The manuscript states, “The current study was foregrounded by the Theoretical Domains Framework (TDF) V2 [21]. This is a theoretical framework that assesses the factors that may enable or militate against the successful implementation of an intervention focused on a specific desired practice change. The TDF consists of fourteen domains developed from 128 theoretical constructs that were synthesized from varieties of behavioural change theories associated with implementation science and practice change…” It could assist readers to identify additional information such as the fourteen domains and those especially relevant to this study, for example.
- **Response-12:** Great comment by the reviewer and we are thankful for this. However. We respectfully submit that the TDF was adequately referenced. However, the mansucript has been revised to provide a summary of the 14 domains of the framework [line 112-116]. We thought this suffices for any interested readers who desire more detailed information about the framework. However

*Atkins L, Francis J, Islam R, et al. A guide to using the theoretical domains framework of behaviour change to investigate implementation problems. Implement Sci. 2017;12:77.*

*Davis R, Campbell R, Hildon Z, Hobbs L, Michie S. Theories of behaviour and behaviour change across the social and behavioural sciences: a scoping review. HealPsychol Rev. 2015;9:323–344.*

*Cadogan C, Ryan C, Francis J, et al. Improving appropriate polypharmacy for older people in primary care: selecting components of an evidence-based intervention to target prescribing and dispensing. Implement Sci. 2015;10:161.*

*Alqubaisi M, Tonna A, Strath A, Stewart D. Quantifying behavioural determinants relating to health professional reporting of medication errors: a cross-sectional survey using the theoretical domains framework. Eur J Clin Pharmacol. 2016;72:1401–1411.*

- **Comment-13:** Lines 140-144. Please see comment above seeking the fourteen domains and those most relevant to this study. It is stated the fourteen domains were grouped into 5 categories, were these grouped by the research team or is this grouping inherent in the TDF? It goes on to say this grouping was influenced by an Irish study but would an Irish study and a Qatari study benefit from the same/similar categorisation? Please explain the research team’s decision-making on this point. Please describe the fourteen domains and the theoretical background as it informs this study. In which geographical region was the framework developed? How does it apply to Qatar.

**Response-13:** Many thanks for this suggestion. The grouping into 5 domains is inherent in the TDF and it was also based on a published Irish study, and we have revised the manuscript to clarify this. The TDF framework is a validated and well documented theoretical framework that has been in several published studies focused on understanding factors associated with behavioral change and implementation science. Relevant references regarding the details of how the framework was developed and validated have been provided in the manuscript. The grouping into 5 domains is inherent in the TDF and it was also based on a published Irish study. The research team adopted these domains because of their well-documented impact on behavioral change irrespective of settings. Additional details regarding the research team decision-making have been provided in line 142-163.

- **Comment -14:** Lines 159-160. For clarity please, does this mean that the responses from the pre-test sample were not included in the dataset of the study for analysis? What happened to them?
- **Response-14:** The pre-test results were discarded and this is in accordance with the established standard in Quantitative survey research.
- **Comment-15:** Lines 170-173. The three research assistants, were these individuals, students, administrative support people or pharmacists? This section could be enhanced by some details about the research assistants since they seem to be the “face” of this study to pharmacist participants. This is a concern because response rates for participation and completion can vary with the characteristics of the person providing the questionnaire. Back in 2002, it was established that even the colour of the paper a printed questionnaire was printed on made a difference, Etter and colleagues, for example, established that pink paper increased response rates by 12%, so provision of detail is important.
- **Response-15:** Many thanks for this important observation. The research assistants were students [3] and the data collection procedure was standardized to minimize any potential bias alluded to in the review comment [line 169-173].
- **Comment-16**: Line 173. The detailed “information about the purpose and anticipated benefits of the study,” how was the information provided? Verbally, written, link to study website? Additional information will assist readers as they consider their own studies.
- **Response-16:** Heartfelt thanks to the reviewer for this comment. The information about the purpose and anticipated benefits of the study was iincluded in the participant consent form given to the respondents to read and sign before the start of data collection. The manuscript has been revised for clarity [line 173-175].

Discussion

- **Comment-17:** Lines 176-177. Please identify by whom they were collected. Research assistants again? The same research assistants for each of the research participants?
- **Response-17:** Please refer to response -15

**Comment-18:** In a manuscript, it is beneficial to identify those functions within SPSS that were used to analyse the data, similarly any recoding of values such as for negatively phrased items. So, was the descriptive function used? Was Factor analysis? If it was then the approach is crucial so PAF, PCA, exploratory? Perhaps Cross-Tabulations might have been used to explore a relationship between two or more variables? More detailed information is needed to understand the results obtained and the conclusions drawn.

**Response-18:** Factor analysis was not done as it was not part of the stated objective. We respectively submit that the information presented in the data analysis suffice. There were no negatively worded items in the data collection tool.

- **Comment-19:** In Methods it was stated that, “…23-item questionnaire comprising three sections including A (demographics = 10 items), B (Enablers = 7 items), and C (Barriers = 6 items). “, so, an expectation would be that how each section was analysed would be clearly identified.

**Response-19:** Thanks to the reviewer. The data analysis details regarding the use of descriptive statistics such as median (IQR) that was used for continuous data with non-normal distribution, and the frequency count (%) for categorical data were adequately provided in line 180-182.

- **Comment-20:** Further, study participants were asked to rank their responses to the items in Section B and C on a 5-point Likert-type scale (highest =5, high = 4, moderate = 3, low = 2, Lowest = 1). However, in Results section Table 2, the columns are labelled as low, moderate and high. If additional analyses or re-coding were undertaken to result in these three categories, that needs to be clearly detailed. Likert data may also be presented as histograms or bar charts where data can be visualised.
- **Response-20:** We are truly grateful for this observation and suggestion. The suggested correction has been done to enhance the clarity of the re-coding into three categories [line 162 – 163].
- **Comment-21:** Lines 173- 176 state, “The respondents who agreed to participate in the study signed the written informed consent form before the start of data collection, and they were all informed of their right to decline participation at any point in the study.” As does Lines 165-167, “The respondents who agreed to participate in the study signed the written informed consent form before the start of data collection, and they were all informed of their right to decline participation at any point in the study.” Please delete one occurrence as these sentences are closely situated and one becomes redundant.
- **Response-21:** Heartfelt thanks to the reviewer for this observation. The repeated sentence has been deleted in line 167. This was an inadvertent error.
- **Comment-22:** The Results section starts with a presentation of the characteristics of the respondents noting for example, that “… [they] consisted mainly of 5 nationalities (91.0%; 182/200) including Indian (40.5%), Egyptian (22.5%), Jordanian (22%) and Sudanese (6%). Since only 182/200 may have responded to this item the absence of Qatari respondents was of interest as in 2024 it might be expected that there could be perhaps some 12 cohorts of graduating students from Qatar University since the course is listed as commencing in 2007 with full accreditation in 2011 (https://www.qu.edu.qa/static_file/qu/colleges/pharmacy/documents/Pharmacy%20A4%20Magazinecompressed%2019AY.pdf).

**Response-22:** We respectfully submit that the results obtained are as presented in the manuscript and the observed trend is consistent with published reports from several studies from Qatar. The community pharmacy sector in Qatar is dominated mainly by foreigners.

- **Comment-23:** The sociodemographic profile of the respondents was featured also in Table 1 which established that data from ten nationalities in total was included. With all nationalities included, the respondent total is 200, the total number of listed respondents. The next results reported are the results for the seven enabling factors and separately the seven factors that act as barriers to community pharmacists’ readiness to implement deprescribing for older adults in Qatar. The items were presented individually, with responses rated as low, medium or high by number of respondents and the per centage that represents. It would be helpful to know what numerical values were represented by the categories, low, medium and high so please provide these. It was helpful to see the item wording and would also have been beneficial in the methodology section because what an item seeks to elicit varies by the wording used. **Response-23:** Many thanks to the reviewer for the excellent suggestions. The numerical values and percentages for each category were presented in the manuscript.
- **Comment-24:** The discussion identified that the community pharmacist respondents would find continuing professional development, the availability of a shared electronic patient record and an improved remuneration package to be key enabling factors in deprescribing in older adults in Qatar who currently reported to be 3.6% of the population. However, there are already reports of an initiative in outpatient pharmacies at Rumailah Hospital, Qatar

(*Alyazeedi, A., Sherbash, M., Algendy, A. F., Stewart, C., Soiza, R. L., Alhail, M., Aldarwish, A., Stewart, D., Awaisu, A., Ryan, C., & Myint, P. K. (2024). Enhancing Medication Safety through Implementing the Qatar Tool for Reducing Inappropriate Medication (QTRIM) in Ambulatory Older Adults. Healthcare (Basel, Switzerland), 12(12), 1186. https://doi.org/10.3390/healthcare12121186*).

If hospital outpatient pharmacies in 2022-23 are using, and others perhaps intending to use Qatar Tool for Reducing Inappropriate Medication (QTRIM) in Ambulatory Older Adults i.e. community dwelling, what is the rationale for community pharmacists to also undertake deprescribing? This considers the significance of the outcomes of the manuscript study compared to an existing tool and implementation in outpatient pharmacies. Please discuss and contextualise also with this statement in the manuscript, “… Furthermore, the identification of the regulatory framework currently guiding community pharmacy practice in Qatar as a key barrier to the implementation of deprescribing in practice is being reported for the first time. This is because the range of clinically-oriented services that community pharmacists are legally permitted to provide are limited…” It may also be helpful to consider for this manuscript, any potential influence of the discussion on inappropriate polypharmacy management as contrasted to deprescribing in a 2023 publication by Zaidan and Awaisu.

**Response-24:** Many thanks to the reviewer. The study alluded to was focused on ambulatory patients in a hospital setting, while the current study is focused on community pharmacists in the community setting. The rationale for community pharmacist to undertake deprescribing is well documented and has been alluded to in the manuscript [Line 67-77].

- **Comment-25:** And here it becomes clear what the reference categories were – see earlier comments. Within all categories, you can see that there is not a clear trend! This should have been reported in the results and been discussed!

**Response-25:** We concur with these great suggestions by the reviewers but they were not reported by the respondents in the current study. However, we think that these are great leads for further research.

- **Comment-26:** It also important to know which town or towns were sampled as different locations may hold different views or contrary views.
- **Response-26:** Many thanks for the comment. All the major municipalities in Qatar were sampled but data collection was done anonymously to protect confidentiality.
- **Comment-27:** It would also be beneficial to have estimates of desired achievements or deliverables, so for example, at year 1, year 3, year 5 and year 10.

**Response-27:** Many thanks to the reviewer for this suggestion. However, this was not part of the objective for the current study. We were focused on the stated study objectives and this suggestion seems like a good lead for future studies in the research area.

- **Comment-28:** Limitations section identifies those characteristics of the study methodology or design that impact the research outcomes or interpretations. As such the limitations are addressed in the section from lines 294 to 301. The limitations identified were firstly, non-probability sampling as participants were drawn predominantly from chain or banner group pharmacies which represent 75% approximately of the community pharmacies in Qatar, however it was not evident in the manuscript which locations were sampling towns or regions as differences in perceptions, experiences and resources might be anticipated in various locations. So, this is a valid issue and benefits from discussion as to any ameliorating factors or processes that the research team may have applied. It was also not known how community pharmacies are owned as the owners may influence pharmacists’ views.
- **Response-28**: This comment has been addressed in response-22 and 26.
- **Comment-29**: The second limitation noted was that a quantitative survey design may have triggered social desirability bias or social acquiescence though the link between methodology (survey) and favourable responses is not clearly explained but benefits from being so.
- **Response**-**29:** Heartfelt thanks to the reviewer for the comment. We respectfully submit that the details regarding the risk of social desirability bias was adequately addressed in line 301-304 in the limitation section.
- **Comment**-**30:** There are thirty-seven references cited in the manuscript of which six are aged (>10 years) with eleven aged within the last five years, for recency. Vancouver style is listed as the expectation of PLOS ONE. This reference (number 36) appears to be inconsistent with Vancouver referencing:

Gerlach N, Michiels-Corsten M, Viniol A, et al. Professional roles of general practitioners, community pharmacists and specialist providers in collaborative medication deprescribing—a qualitative study. BMC Fam Pract. 2020/09/06 ed. 2020 Sep 4; 21(1):183.

Please review the reference list for consistency with style.

- **Response**-**30:** Massive thanks to the reviewer for this observation. The references have been reviewed for consistency.

**Reviewer #2:**

- **Comment-1**: rewrite the title as the title is not clear such using facilitators instead of enablers.
- **Response-1**: Heartfelt thanks to the reviewer for the suggestion. We respectfully submit that the term “enablers” is clear and has been used in several published studies focused on factors associated with behavioral change and implementation science.
- **Comment-2:** I prefer to attach the developed questionnaire in the appendix or supplement file
- **Response**-**2:** Heartfelt thanks to the reviewer for the suggestion and we fully the reviewer’s preference. The data collection tool that we used is available on a reasonable request.
- **Comment**-**3:** I think also mention how did you summarize answers from five choices to three choice (table 2) in the methodology section after line 162-163
- **Response-3:** This comment has been addressed in our response-20 to comment-20 from referee-#1
- **Comment-4:** I think mention the current practice in Qatar, it is manly traditional practice or there is/are some advance practice such as reviewing medication, providing vaccination, prescribing authorizes, etc... will be beneficial and strengthen the study.
- **Response-4:** Heartfelt thanks to the reviewer for the suggestion. However, we were desirous of being laser-focused on the study objective and this was why we thought we should focus mainly on the fact that the provision of a deprescribing service guided by a structured framework is currently not within the scope of practice for community pharmacists generally in the Middle Eastern Gulf countries [line 101-103].
