# Supplementary material for: Enablers and barriers to community pharmacists’ readiness to implement deprescribing of inappropriate medications for older adults in Qatar
Source: PLoS One. 2025 Jan 30;20(1):e0316363. doi: 10.1371/journal.pone.0316363 (PMC11781633; doi:10.1371/journal.pone.0316363)
Supplement: Attachment — Submitted filename: Response_2 to Reviewers_2nd Revision.docx [file pone.0316363.s003.docx]

07 December 2024

The Editor-In-Chief

PLOS ONE

Dear Sir,

Re: Manuscript #PONE-D-24-26224R1 – “Enablers and barriers to community pharmacists’ readiness to implement deprescribing of inappropriate medications for older adults in a developing setting”

We are sincerely grateful for the opportunity to revise, for the second time, the manuscript #PONE-D-24-26224R1, titled “Enablers and barriers to community pharmacists’ readiness to implement deprescribing of inappropriate medications for older adults in a developing setting” which is under your consideration for publication in the PLOS ONE. We thankful for the additional comments and useful suggestions provided by the reviewers. Please find stated below our point-by-point response to the reviewers’ comments.

**REVIEWER’S COMMENTS**

**Reviewer #1:**

- **Comment-1:** Currently those in Qatar older than sixty years of age constitute 3.6% of the population though that is predicted to increase to approximately 20.3% by 2050, which is in 26 years’ time, so lacking apparent immediate urgency. So, please seek to firmly and clearly identify the significance or implications of undertaking this study now in 2024 - Why is it needed? Why now? What has to change and what else has to happen for the activity on which this research is focused to come into effect. In my experience, often authors may be well aware of what is happening in their setting, what could be desirable and will of the pharmacist population to provide a proposed service or modify a standard procedure, for example. However, readers from outside the setting are often quite unfamiliar with a study's setting, and in lieu of more information may wonder if it is much the same as in the reader's setting. In my experience providing context, information, including some statistics will assist the reader the view the study within the actual study setting.
- **Response-1:** We are thankful once again for this important observation and we see the points raised by the reviewer. However, we thought we have addressed this concerns in our previous response. It is generally well-documented that prevention is not only better but it is more cost-effective than cure. Hence, we are of the opinion that the potential clinical and financial burden associated with the harms inherent in the use of inappropriate medications in elderly population is better avoided by provision of deprescribing service especially at the primary care level where community pharmacists are easily accessible. In addition, 3.6% of the current population translates to about 120,000 elderly patients in Qatar (Estimated pop is about 3million). Hence, there is a lot of potential inherent benefits associated with the deployment of deprescribing of inappropriate medications in one of the most vulnerable patient group such as the elderly without having to wait unnecessarily until 2050. In addition, it is generally well established that early deployment of interventions focused improving patient outcomes and strengthening the health system often result lasting positive impact on public health.
- **Comment-2:** Issue - A little more clarity would beneficial - what is a dispassionate review? What benefit arises from a dispassionate view? Why not a standard protocol? Why not take into consideration the patient’s perceptions of their medications or issues they may be having? Further, the key is to identify medications that are no longer required - for which reasons? duplication of therapy? continuation of medication to manage an acute condition? removal of a medication that manages an adverse effect of another medication taken by the patient? Issue- benefit of additional clarity as to why deprescribing needs to be added, legally, to community pharmacists’ scope, and why the timing since hospital pharmacists have and are trialing a deprescribing initiative- how is it that deprescribing is within scope for hospital pharmacists in Qatar but not community pharmacists?

**Response-2**: Heartfelt thanks to the reviewer for this additional comment. We submit with all due respect that the word “dispassionate” is self-explanatory. However, we have replaced this word with a more appropriate one as suggested [Pg 4, line 64]. In addition, we thank the reviewer for all the other issues that was raised in the review comment. However, we submit with all due respect these issues are outside the scope of the objectives of the current study. However, we believe that they are good leads for further research, and we are thankful to the reviewer.

**Comment-3:** Lines 63-65 assert that the greatest risk to the elderly from medication lies in the number of medicines, and certainly, the more medicines taken, the more there can be a risk of drug-drug, drug-herb, drug-disease state interactions, but there are also some medicines that have an inherent risk of adverse effects in the elderly, even with a lesser number of medications taken. These include NSAIDS, medicines with an anticholinergic effect, some diuretics, antihypertensives and others (<https://www.msdmanuals.com/en-au/professional/geriatrics/drug-therapy-in-older-adults/drug-categories-of-concern-in-older-adults>). So some additional clarity in the definition about how medicines may be harmful as medicines have many different perceptions to lay people, to various health professionals such as doctors, specialist doctors, nurses, pharmacists.

**Response-3:** Heartfelt thanks to the reviewer for this excellent suggestion. We concur and the manuscript has been revised accordingly [Pg 3, line 61-63].

- **Comment-4:** Lines 102-104. The manuscript states, “Indeed, only one such study focused on the assessment of community pharmacists’ knowledge of deprescribing and their self-perceived enablers and barriers to providing the service in the United Arab Emirates was identified.”

It can be helpful to identify the search criteria utilised as the databases, the search terms (e.g. whether words were used or truncation was included) and the timeframe as search engines often give different outcomes at different times, so when the search occurred also matters.  **Response-4:** Many thanks to the reviewer for the suggestion. However, the study was not a systematic review and hence we are of the opinion that the information presented about the procedure used for the literature review suffices.

- **Comment-5:** METHODS: The requested information in PLOS ONE is detailed and clear – the journal requires sufficient detail to allow suitably skilled researchers to fully replicate this study. When the methods are well established, authors may cite articles where those are described in detail, but even so THIS manuscript should include sufficient information to be understood independent of those references. Please provide a copy of the survey in an appendix so format, item wording, response sets, item order can be considered., so clarity in this study’s use is beneficial
- **Response-5:** Heartfelt thanks to the reviewer for this comment. Adequate information has been provided in the methods sections regarding all the procedures used at the various stage of the study, including the development and validation of the data collection tool. The data collection tool is available upon reasonable request.
- **Comment-6:** Lines 63-65 assert that the greatest risk to the elderly from medication lies in the number of medicines, and certainly, the more medicines taken, the more there can be a risk of drug-drug, drug-herb, drug-disease state interactions, but there are also some medicines that have an inherent risk of adverse effects in the elderly, even with a lesser number of medications taken. These include NSAIDS, medicines with an anticholinergic effect, some diuretics, antihypertensives and others (https://www.msdmanuals.com/enau/professional/geriatrics/drug-therapy-in-older-adults/drug-categories-of-concern-in-older-adults).
- **Response-6:** Sincere thanks to the reviewer for this excellent comment. We concur with the reviewer point and we think this assertion has already been captured in the manuscript [Line 61-65]. We were conscious of balancing comprehensiveness with conciseness to minimize redundancies.
- **Comment-7** ANALYSIS: The manuscript identifies that IBM SPSS (Statistics for Windows, Version 29.0. Armonk, NY: IBM Corp.) software was used for data analysis. Descriptive statistics such as median (IQR) was used for continuous data with non-normal distribution while frequency (%) was used for categorical data, with significance set at p ≤0.05. Additional clarity sought: In a manuscript, it is beneficial to identify those functions within SPSS that were used to analyse the data. In survey research, it usually matters both what you ask and how you ask it. So, for example, the manuscript data was stated to have been analysed to yield descriptive statistics such as median (IQR) for continuous, non-normal data and frequency for categorical data. The SPSS Descriptive function data analysis can yield mean, sum, standard deviation, variance, minimum, maximum, range, skewness, kurtosis and standard error of the mean. The SPSS Frequencies function yields mean, standard deviation, variance, minimum, maximum, range, skewness, kurtosis, valid & missing responses, median, mode quartiles, percentiles. It is beneficial to identify the SPSS function actually used as that assists a reader to see whether that data analysis could yield the results of the study or whether a different analysis may have yielded more relevant or more useful result. Words discussing the analysis can also assist readers as can the actual wording of items, order of items, response set and format of the survey, the study instrument. The manuscript identified that it elicited responses on a Likert-like response set. Most commonly Likert scales have two extreme responses, two moderate responses and a neutral response per item (strongly disagree, disagree, neutral, agree, strongly agree or reverse order). Subsequently, the responses were described as ranked, “Further, study participants were asked to rank their responses to the items in Section B and C on a 5-point Likert-type scale (highest =5, high = 4, moderate= 3, low = 2, Lowest = 1)”. The difference between rating and ranking lies in the information elicited. Rating scale questions such as Likert scales seek to elicit respondents’ attitude(s) towards something (how much do you agree with this statement, for example). A ranking question seeks a respondent’s preference order. More detailed information is needed to properly understand the results obtained and the conclusions drawn, so please provide additional information, and in particular, more detail.

**Response-7:** We thank the reviewer for these comments. We respectfully submit that adequate information has been provided with sufficient clarity in the methods sections regarding the data analyses conducted in the study [Line 141-184]. We are of the opinion that this suffices and it is consistent with the established norm in the scholarly world.

- **Comment-8:** RESULTS: PLOS ONE accepts single categories across Results/Discussion/Conclusions or merged categories such as Results/Discussion or Discussion/ Conclusions. This manuscript presents all three sections separately. Only Results will be addressed for review in this section. The Results section starts with a presentation of the characteristics of the respondents noting for example, that “… [they] consisted mainly of 5 nationalities (91.0%; 182/200) including Indian (40.5%), Egyptian (22.5%), Jordanian (22%) and Sudanese (6%). Since only 182/200 may have responded to this item the absence of Qatari respondents was of interest as in 2024 it might be expected that there could be perhaps some 12 cohorts of graduating students from Qatar University since the course is listed as commencing in 2007 with full accreditation in 2011 (https://www.qu.edu.qa/static_file/qu/colleges/pharmacy/documents/Pharmacy%20A4%20Magazine-compressed%2019AY.pdf). Additional detail and context would be helpful to fully understand the results of the study and its outcomes. It would be beneficial to understand where Qatari educated pharmacists work – data were elicited from chain pharmacies and none were identified as Qatari, so perhaps Independent pharmacies? Industrial pharmacy? Hospital pharmacy? Further, in a 2021 study which included independent and chain pharmacies differences were noted such as a preponderance of males rather than the females majority identified in the current study. Response-22: We respectfully submit that the results obtained are as presented in the manuscript and the observed trend is consistent with published reports from several studies from Qatar. The community pharmacy sector in Qatar is dominated mainly by foreigners. Firstly, then please provide the context using the published reports from several studies in Qatar cited in your reply as response 22. Irrespective, pharmacists working in Qatar would seem to be bound by the Law irrespective of nation of initial education and registration/authorisation to practice. Further, it is of interest to pharmacists/researchers globally where Qatari educated pharmacists work and WHY community pharmacy is run by foreigners, which may often be considered an issue in some nations. PLOS is an international journal and while pharmacy has a role in most if not all nations, in my experience, the roles, ownership, ability to practice as an out-of-nation educated pharmacist vary, sometimes greatly.
- **Response-8:** We are thankful to the reviewer for this comment and we see the point. However, we thought we have addressed this in our previous response. In addition, we respectfully submit that some of the suggested corrections are outside the scope of the stated study objectives. We sincerely appreciated the reviewer’s generosity in identifying these potential leads for further research but we desirous of ensuring that the manuscript is laser-focused on the stated objectives.

**Comment-9** - DISCUSSION: The discussion identified that the community pharmacist respondents would find continuing professional development, the availability of a shared electronic patient record and an improved remuneration package to be key enabling factors in deprescribing in older adults in Qatar who currently reported to be 3.6% of the population. However, there are already reports of an initiative in outpatient pharmacies at Rumailah Hospital, Qatar (Alyazeedi, A., Sherbash, M., Algendy, A. F., Stewart, C., Soiza, R. L., Alhail, M., Aldarwish, A., Stewart, D., Awaisu, A., Ryan, C., & Myint, P. K. (2024). Enhancing Medication Safety through Implementing the Qatar Tool for Reducing Inappropriate Medication (QTRIM) in Ambulatory Older Adults. Healthcare (Basel, Switzerland), 12(12), 1186. https://doi.org/10.3390/healthcare12121186). If hospital outpatient pharmacies in 2022-23 are using, and others perhaps intending to use Qatar Tool for Reducing Inappropriate Medication (QTRIM) in Ambulatory Older Adults i.e. community dwelling, what is the rationale for community pharmacists to also undertake deprescribing? This considers the significance of the outcomes of the manuscript study compared to an existing tool and implementation in outpatient pharmacies. Please discuss and contextualize.

**Response-9** - Many thanks to the reviewer for this comment. This comment has been addressed in our previous response as stated below, and we believe that this suffices:

**“***The study alluded to was focused on ambulatory patients in a hospital setting, while the current study is focused on community pharmacists in the community setting. The rationale for community pharmacist to undertake deprescribing is well-documented and has been alluded to in the manuscript [Line 68-79].*

**Comment-10:** For example, how many elderly people see only community pharmacists, how many or what proportion see both a hospital pharmacist and a community pharmacist and how many or what proportion see only a hospital pharmacist? Some of the reasons that detail and information is important to understanding is what others say about pharmacy practice in Qatar. For example, Nadir Kheir in a book chapter in December 2016 entitled "Pharmacy Practice in Qatar" identified that pharmacy services in Qatar are rapidly developing and pharmacists are rapidly changing, committing to lifelong learning, self leadership and self development. Further, within that chapter it was observed that one of the strongest drivers of the change and development in pharmacy practice is Qatar's National Health Strategy which a further on community pharmacy. It was envisioned that community pharmacy could contribute to a primary health care model of practice and care. So, from 2016 to 2024, what has happened?

**Response-11:** Many thanks for these interesting suggestions. However, this is outside the scope of the stated objectives for the current study. We strongly believe that these are great leads for further research.

- **Comment-12:** LIMITATIONS: Limitations section identifies those characteristics of the study methodology or design that impact the research outcomes or interpretations. As such the limitations are addressed in the section from lines 294 to 301. The limitations identified were firstly, non-probability sampling as participants were drawn predominantly from chain or banner group pharmacies which represent 75% approximately of the community pharmacies in Qatar. So, this is a valid issue and benefits from discussion as to any ameliorating factors or processes that the research team may have applied. It was also not known how community pharmacies are owned as the owners’ views and actions may also influence employed pharmacists’ perceptions. Context is important as there is not one single vision and practice of pharmacy, or even community pharmacy, world-wide. How different might community pharmacy be in New Zealand, in Thailand, in Japan, in Spain, in Sweden, in Brazil, for example? In some countries, pharmacies may only be owned by registered pharmacists, sometimes with limits imposed on the number of pharmacies owned by a registered pharmacist, in some countries, companies can own pharmacies and employ pharmacists to provide services, so context benefits from greater clarity about the situation in Qatar. The second limitation noted was that a quantitative survey design may have triggered social desirability bias or social acquiescence though the link between methodology (survey) and favourable responses is not clearly explained but benefits from being so. To my perception, this is a well published area - there are a number of approaches such as published articles that consider this and also social desirability scales which could be included in the study instrument, and other options that can be explored such as Lechner and colleagues 2019; Bergen and Labonte 2020; Larson 2019; Primi and colleagues 2019; Kreitchmann and colleagues 2019; Tan and colleagues 2022 . Which approaches did the researchers use? If none of the approaches were used what was the rationale for not doing so?
- **Response-12:** Great comment by the reviewer and we are thankful for this. However. We respectfully submit that the detailed information about the strengths and limitations of the study have been provided with sufficient clarity in the manuscript, and this is consistent with the established scholarly norms in the research area [Line 293 – 303]. In addition, the issue relating community pharmacies ownership structure and how these might influenced community pharmacists’ perception have been addressed in the discussion of one of the study findings that showed that community pharmacists’ focus on meeting organizational sales target was identified as a top-ranked barrier to readiness to implement deprescribing, and that this was being reported for the first time, and seems to suggest a dominant focus on the business side rather clinical practice in the community pharmacy sector in Qatar [Line 208-290].

**Reviewer #2:**

- **Comment-1**: The manuscript looks good and I agree with proceeding for publication, but small comment; the sentence in the line 99-101, I did not any references of the studies that were mentioned
- **Response-1**: Heartfelt thanks to the reviewer for the kind comments and suggestion. We did not cite any reference to support that the provision of a deprescribing service guided by a structured framework is currently not within the scope of practice for community pharmacists generally in the Middle Eastern Gulf countries because no published study has reported this. The current will be the first to do so, hopefully if it is finally accepted for publication in the prestigious PLOS ONE.
